# Terfenadine resensitizes doxorubicin activity in drug-resistant ovarian cancer cells *via* an inhibition of CaMKII/CREB1 mediated ABCB1 expression

**DOI:** 10.3389/fonc.2022.1068443

**Published:** 2022-11-10

**Authors:** Wei Huang, Shu Yang, Yu-Shan Cheng, Ni Sima, Wei Sun, Min Shen, John C. Braisted, Weiguo Lu, Wei Zheng

**Affiliations:** ^1^ Women’s Hospital, School of Medicine, Zhejiang University, Hangzhou, China; ^2^ National Center for Advancing Translational Sciences (NCATS), National Institutes of Health (NIH), Bethesda, MD, United States; ^3^ Women’s Reproductive Health Research Laboratory of Zhejiang Province, Women’s Hospital, School of Medicine, Zhejiang University, Hangzhou, China

**Keywords:** drug-resistant, ovarian cancer, terfenadine, CaMKII, doxorubicin

## Abstract

Ovarian cancer is one of the most lethal gynecological malignancies. Recurrence or acquired chemoresistance is the leading cause of ovarian cancer therapy failure. Overexpression of ATP-binding cassette subfamily B member 1 (ABCB1), commonly known as P-glycoprotein, correlates closely with multidrug resistance (MDR). However, the mechanism underlying aberrant ABCB1 expression remains unknown. Using a quantitative high-throughput combinational screen, we identified that terfenadine restored doxorubicin sensitivity in an MDR ovarian cancer cell line. In addition, RNA-seq data revealed that the Ca2+-mediated signaling pathway in the MDR cells was abnormally regulated. Moreover, our research demonstrated that terfenadine directly bound to CAMKIID to prevent its autophosphorylation and inhibit the activation of the cAMP-responsive element-binding protein 1 (CREB1)-mediated pathway. Direct inhibition of CAMKII or CREB1 had the same phenotypic effects as terfenadine in the combined treatment, including lower expression of ABCB1 and baculoviral IAP repeat-containing 5 (BIRC5, also known as survivin) and increased doxorubicin-induced apoptosis. In this study, we demonstrate that aberrant regulation of the Ca2+-mediated CAMKIID/CREB1 pathway contributes to ABCB1 over-expression and MDR creation and that CAMKIID and CREB1 are attractive targets for restoring doxorubicin efficacy in ABCB1-mediated MDR ovarian cancer.

## Introduction

Ovarian cancer is one of the worst cancers and the major cause of mortality among gynecologic tumors (1). Due to the absence of clear early-stage symptoms, more than 75% of ovarian cancer patients are diagnosed at an advanced stage, with a 5-year survival rate of 20% (2–4). Currently, debulking surgery followed by chemotherapy is the usual treatment for ovarian cancer in an advanced stage (5). However, the development of tumor resistance during treatment is common and poses challenges in ovarian cancer therapy (6, 7). Indeed, over 80% of cases respond to first-line treatment, yet 70% of patients experience cancer recurrence within the first three years (8).

Overexpression of ATP binding cassette subfamily B member 1 (ABCB1) is a well-known molecular mechanism responsible for multidrug resistance (MDR) in malignancies such as ovarian cancer (6, 9, 10). ABCB1, also known as P-glycoprotein 1 (P-gp), is an ATP-driven efflux transporter that pumps substrates from cells. To protect organs from toxins, it is abundantly distributed in the blood-brain barrier, placenta, kidneys, and intestines (11). Numerous anticancer medicines, including doxorubicin, vincristine, paclitaxel, anthracyclines, and taxanes, are ABCB1 substrates (10, 11). Hence, overexpression of ABCB1 in cancer cells decreases intracellular concentrations of these drugs and produces MDR (12). Since co-administration of an effective ABCB1 modulator with anticancer drugs was deemed to be a viable strategy for overcoming ABCB1-mediated MDR malignancies, efforts have been made to generate ABCB1 inhibitors in the past few decades. Despite the fact that numerous ABCB1 inhibitors have been developed, their clinical translation has been limited due to their low binding affinities, excessive toxicity, or non-specificity (13, 14), indicating the need for new ABCB1 transporter inhibitors or strategies to overcome the MDR caused by ABCB1 overexpression.

Terfenadine is a histamine receptor H1 (HRH1) antagonist that was once employed to treat allergy disorders. Recent studies have demonstrated that terfenadine inhibits cell growth and induces apoptosis in neoplastic mast cells, melanoma cells, and breast cells *via* altering intracellular calcium homeostasis, caspase activation, and the mitochondrial pathway (15–19). Moreover, a synergistic effect of terfenadine and anticancer drugs has been demonstrated in the treatment of breast cancer and lung cancer (20, 21). However, it is unknown how terfenadine functions in this combinational therapy. Intriguingly, terfenadine has been related to a decrease in calcium influx caused by L-type calcium channels (LTCC) activation in rat and human cells (22, 23), showing terfenadine can regulate intracellular calcium homeostasis. Calcium works as a second messenger in cells to activate the downstream RNA polymerase to trigger gene transcription, which is involved in various cellular processes, such as cell division, proliferation, *etc.* (24, 25). Ca^2+^ signaling alterations are linked to carcinogenesis, tumor development, and metastasis (26). Moreover, it has been found that calcium signaling is connected with drug resistance. Activation of transient receptor potential channels, for instance, is associated with chemoresistance in a number of malignancies (27).

In this work, using quantitative high-throughput combinational screening (qHTCS), we found that terfenadine reverses doxorubicin resistance in MDR ovarian cancer cells. In addition, we demonstrate that terfenadine interacts directly with calcium/calmodulin dependent protein kinase II delta (CAMK2D) and inhibits the subsequent ectopic activation of the CAMK2/cAMP responsive element binding protein 1 (CREB1) pathway in an ABCB1-mediated MDR ovarian cancer line, A2780-ADR. In fact, either the CAMK2 or CREB1 inhibitor resensitizes doxorubicin-resistant ovarian cancer cells, showing that the CAMK2/CREB1 pathway is a suitable target pathway for future therapeutic development.

## Materials and methods

### Compounds and antibodies

Terfenadine was purchased from Sigma-Aldrich (catalog number: T9625). Topotecan, paclitaxel, KN62, and KN93 were obtained from Selleck Chemicals (catalog number: S1231, S1150, S7422, and S7423). Rhodamine 123 was purchased from MedChemExpress (catalog number: HY-D0186) Antibodies used in experiments are listed in **Table S1**.

### Cell culture

All the human ovarian cancer cell lines were purchased from Sigma-Aldrich. Cells were cultured in RPMI 1640 medium with 10% fetal bovine serum (FBS) and 100 U/mL penicillin-streptomycin at 37°C with 5% CO_2_.

### Quantitative high-throughput combinational screening

ATP content assay (Promega) was conducted according to the manufacture’s protocols. Briefly, A2780-ADR cells were plated at 500 cells/well in 5 µL of RPMI 1640 medium with 10% FBS and 100 U/mL penicillin-streptomycin in white, solid-bottom 1,536-well plates and incubated 4 h at 37°C. Four concentrations of compounds from the library of pharmacologically active compounds (LOPAC, Sigma-Aldrich) consists of 1,280 small molecules, the NIH Chemical Genomics Center Pharmaceutical (NPC) collection with 4,265 compounds (28), as well as the Mechanism Interrogation Plate (MIPE) with 1,920 compounds were added to assay plates at 23 nL/well using a pintool station (WAKO Scientific Solutions, San Diego, CA). After a 72-h incubation at 37°C with 5% CO_2_, the mixture of ATP LITE assay reagents was added to the assay plates at 5 µL/well. After incubation for the indicated time, the luminescence signal in the plates were detected using a ViewLux plate reader (PerkinElmer).

### Rhodamine123 accumulation assay

A2780-ADR cells were seeded onto 96-well plates at a density of 5,000 cells/well. The cells were pretreated with 2.5 to 10 µM terfenadine for different time. After pretreatment, the cells were incubated with 5 µM Rhodamine123 (Rh123) in culture medium and kept in the dark at 37°C with 5% CO_2_ for 60 min. Plates were then washed twice with pre-warmed PBS, filled with 100 µl/well PBS, and measured using a Tecan reader at 485 nm excitation and 535 nm emission.

### Caspase activity assay and ATP content cell viability assay

Caspase-3/7 activity assay (Caspase-Glo, Promega) and ATP content cell viability assay (CellTiter-Glo, Promega) were conducted according to the manufactures’ protocols. Ovarian cancer cells were plated at 3,000 to 5,000 cells/well in 100 µL of complete culture medium in white, solid-bottom 96-well plates and incubated overnight at 37°C with 5% CO_2_. Compounds were added to the assay plates at indicated concentrations at 100 µL/well diluted in medium. After a 24 h (caspase 3/7 assay and ATP content cell viability assay) incubation at 37°C with 5% CO_2_, the mixtures of assay reagents at 100 µL/well were added to the assay plates. After incubation for the indicated times from the protocols, the luminescence signal in assays plates were detected in a ViewLux plate reader.

### RNA-sequencing analysis

RNA-sequencing analysis of A2780 and A2780-ADR was performed by Q2 Solutions as previously described (29, 30). RNA was isolated by Qiagen miRNeasy Mini Kit. cDNA libraries were generated using Illumina TruSeq Stranded mRNA sample preparation kit (Illumina # RS-122-2103). Read counts of each sample were normalized with DESeq and ran a negative binomial two sample test to find significant genes in higher transcript abundance in either sample. RNA sequencing data have been deposited in Gene Expression Omnibus (GEO) under accession number GSE177038.

### Western blotting

Cells were lysed in RIPA buffer (Cell Signaling Technology) supplemented with protease inhibitors (cOmplete ULTRA Tablets, EDTA-free, Roche) and phosphatase inhibitor cocktail (PhosSTOP, Roche). The cell lysates were centrifuged at 16,000 rpm for 30 min. Supernatant was collected for protein quantitation with a BCA assay kit (Pierce BCA Protein Assay Kit, Thermo Fisher Scientific). The supernatant with similar protein concentrations were subsequently applied to Bis-Tris or Tris-Acetate gels for protein separation. Proteins were transferred to polyvinylidene difluoride (PVDF) membrane by dry transfer (iBlot 2 Gel Transfer Device, both from Thermo Fisher Scientific) or tank wet transfer. Immunoblot analysis was performed with indicated antibodies and the chemiluminescence signal was visualized with Luminata Forte Western HRP substrate (EMD Millipore) in a BioSpectrum system (UVP, LLC). The chemiluminescence intensity of the band was calculated in the VisionWorks LS software (UVP, LLC).

### Cellular thermal shift assay

CETSA was performed as previously described (31). A2780-ADR cells were harvested, rinsed with PBS, and re-suspended in detergent-free buffer (25 mM HEPES pH 7.0, 20 mM MgCl_2_, 2 mM DTT) supplemented with protease inhibitors and phosphatase inhibitor cocktail. The cell suspensions were lysed *via* three freeze-thaw cycles with liquid nitrogen. The cell lysates were centrifuged at 16,000 rpm for 20 min at 4 °C to pellet the cell debris from the soluble fraction. The soluble portion were diluted in detergent-free buffer and divided into two aliquots, with or without 600 μM terfenadine treatment. After 60 min incubation at room temperature, each sample was divided into 12 small aliquots in 50 μL/tube and individually heated at different temperatures (37 to 70 °C with 3 °C interval) for 3 min in a thermal cycler (Eppendorf), followed by immediate 3 min cooling cycle on ice. The heated samples were centrifuged at 20,000 × g for 20 min at 4 °C to remove the precipitates from the soluble fractions. The supernatant was examined by western blotting with CAMKII antibody. The relative chemiluminescence intensity of each sample at different temperatures was used to plot the temperature dependent melting curve. The apparent aggregation temperature (T_agg_) was calculated by nonlinear regression. The statistically significance between two curves were analyzed by extra sum-of-squares F test. All data represent mean ± SEM of at least 3 replicates.

### Data analysis

The primary screen data were analyzed using customized software developed internally (32). All data from the cell-based assays were presented as the mean ± standard error of the mean (SEM) with at least three independent experiments unless otherwise stated. Half maximal inhibitory concentrations (IC_50_) of doxorubicin or compounds were calculated using Prism software (version 7, GraphPad Software, San Diego, CA). All imaging data are presented as the mean ± SEM and represent data from cells in at least 10 fields from three or more independent experiments. The two-tailed unpaired Student’s test of the mean was used for single comparisons of statistical significance between experimental groups. One-way analysis of variance (ANOVA) with Bonferroni test was used for multiple comparisons. Bliss independence with Prism or SynergyFinder (33) was used to define synergistic or additive effects.

## Results

### Terfenadine restores doxorubicin activity to MDR ovarian cancer cells

**Figure 1:**
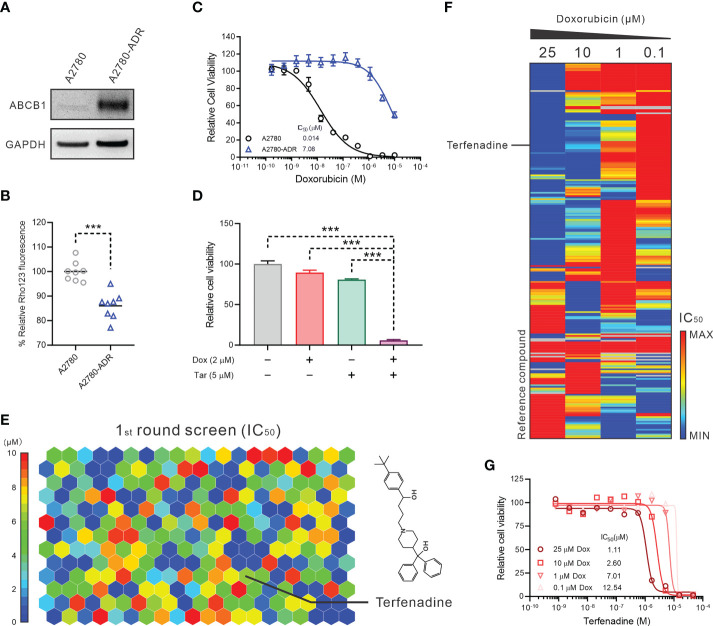
Terfenadine restores the activity of doxorubicin in MDR ovarian cancer cells. **(A)** Western blot analysis of ABCB1 in multidrug resistance (MDR) ovarian cancer cells A2780-ADR and its parental cell A2780. A representative image was shown here. **(B)** Rho123 accumulation in A2780-ADR and its parental cell A2780. **(C)** Dose-response curves of doxorubicin in MDR ovarian cancer cells A2780-ADR and its parental cell A2780. **(D)** Cell viability of MDR ovarian cancer cells A2780-ADR treated with 2 μM doxorubicin (Dox), 5 μM tariquidar (Tar), or both for 48 hours. DMSO was used as a non-treated control. **(E)** The heatmap shows 246 compounds were identified that efficiently inhibited the proliferation of A2780-ADR cells as monotherapy in the first round of screening. The color represents the IC_50_ of each compound, as the scale bar showed. **(F)** The heatmap shows enrichment of A2780-ADR for a strong response to specific drug categories (columns) combined with doxorubicin (rows). Drug-category-response scores are based on IC_50_ (μM). **(G)** A2780-ADR dose-response curves to terfenadine in the presence of 0.1, 1, 10, and 25 μM doxorubicin (Dox). Statistical analysis was performed using a two-tailed t-test. ***p<0.001.

To explore potential novel therapies for ABCB1-mediated MDR ovarian cancer cases in the clinic, we conducted a qHTCS against an ABCB1-overexpressing MDR ovarian cancer cell line, A2780-ADR. Compared to the parental A2780 cells, the A2780-ADR cells exhibited a higher expression and overall activity of ABCB1, as demonstrated by a considerable rise in protein level in Western blot detection and a significantly reduced cellular accumulation of Rho123, an ABCB1 substrate (**Figures 1A, B** and **Figure S1A**). The IC_50_ values of three ABCB1 substrates (doxorubicin, topotecan, and paclitaxel) for the A2780-ADR cells were 7.08 uM, 0.0081 uM, and 0.88 uM, which were significantly higher than 14 nM, 0.95 nM, and 0.0016 nM, respectively, for the A2780 cells (**Figure 1C** and **Figures S1B, C**). In the presence of tariquidar, a specific ABCB1 inhibitor, their anticancer effectiveness against A2780-ADR cells also increased (34) (**Figure 1D** and **Figures S1D–F**).

The qHTCS was performed in two stages. In the first stage, we examined 6,016 pharmacologically active compounds as single drugs at five doses in a luminescent cell viability assay to narrow down the compound pairs. 246 compounds were found that effectively inhibit the proliferation of A2780-ADR cells with an IC_50_ < 10 µM (**Figure 1E**). To further identify compounds that showed combination effects with doxorubicin, these 246 compounds were evaluated at 11 concentrations in combination with four doxorubicin concentrations at 0.1, 1, 10, and 25 µM, separately. Consequently, 24 compounds were identified as doxorubicin synergistic compounds in A2780-ASR cells, as indicated by the decreasing IC_50_ of each drug as the doxorubicin dose rose (**Figure 1F** and **Table S2**). Terfenadine was selected for further investigation (**Figure 1G**) since the mechanism of terfenadine and doxorubicin combination is unknown and terfenadine’s anticancer activity has been described (15, 16).

### Terfenadine resensitizes doxorubicin-induced apoptosis in MDR ovarian cancer cells

**Figure 2:**
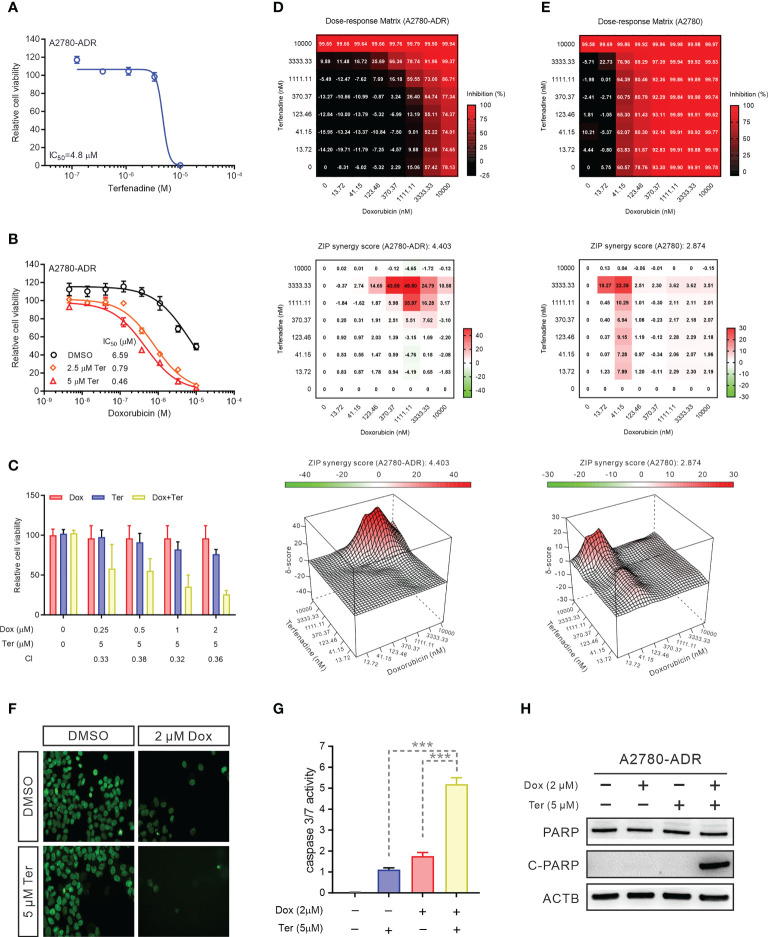
Terfenadine restores doxorubicin-induced apoptosis in MDR ovarian cancer cells **(A)** Terfenadine dose-response curves of MDR ovarian cancer cells. **(B)** Dose-response curves for doxorubicin in MDR ovarian cancer cells in the absence or presence of terfenadine (Ter). **(C)** A Bar graph showing the synergistic effects of terfenadine (Ter) and doxorubicin (Dox) on MDR ovarian cancer cells. Calculated CI values are presented below the plots. **(D, E)** Synergy matrixes (bottom) and surface plots (top) show the synergy between doxorubicin and terfenadine on A2780 **(D)** and A2780-ADR **(E)** cells (n = 3). **(F)** Nuclear staining of MDR ovarian cancer cells treated with the indicated concentration of doxorubicin (Dox), terfenadine (Ter) or both. DMSO was used as a non-treated control. **(G)** Caspase3/7 activity in MDR ovarian cancer cells treated with the indicated concentration of doxorubicin (Dox), terfenadine (Ter) or both. DMSO was used as a non-treated control. **(H)** Western blot analysis of PARP in MDR ovarian cancer cells after treated with 2 μM doxorubicin (Dox), 5 μM terfenadine (Ter), or both for 24 h. ACTB was used as the loading control. All values represent the mean ± SEM (n = 3 replicates). Western blot images were shown as one of three repeated experiments. Statistical analysis was performed using two tailed t-test (*** *p <* 0.001).

With an IC_50_ of 4.8 µM, the inhibitory activity of terfenadine as a single agent was confirmed in MDR A2780-ADR cells (**Figure 2A**). As evidenced by the shifted toxicity curve in MDR cells, the combined treatment significantly decreased the IC_50_ of doxorubicin in a dose-dependent manner, indicating the potential synergistic effect of these two drugs (**Figure 2B**).

To quantify these enhanced anticancer effects, we computed the combinational index (CI) (CI<1, synergism; CI=1, additive; CI>1, antagonism) (35). The mean CI value was 0.35, showing that the interaction in A2780-ADR cells is synergistic (**Figure 2C**). To further evaluate the synergism and determine the best synergistic concentration, effects were investigated using a dose-response matrix and analyzed using the zero interaction potency (ZIP) model (33, 36). As a result, terfenadine exhibited a synergistic effect with doxorubicin. The average ZIP synergy scores for A2780 cells and A2780-ADR cells were 2.874 and 4.403, respectively (**Figures 2D, E**). Notably, in resistant cells, the synergy score reached 49.80 when resistant cells were treated with 3.33 µM terfenadine and 1.11 µM doxorubicin, which was higher than the highest score of 22.39 in sensitive cells, showing a greater synergistic effect in MDR cells than in their parental cells, indicating that terfenadine will target the abnormally activated pathway associated with ABCB1 overexpression in MDR cells. In addition, the combination therapy lowered cell counts in a nuclear staining-based counting assay (**Figure 2F**), enhanced caspase-3/7 activity (**Figure 2G**), and promoted PARP cleavage (**Figure 2H** and **Figure S2A**), suggesting that the A2780-ADR cells were induced to undergo apoptosis. During the expanded testing, terfenadine had a comparable effect on the toxicity of paclitaxel and topotecan for A2780-ADR cells (**Figures S2B, C**).

### Neither hERG nor H1R were the functional targets of terfenadine in the combination

**Figure 3:**
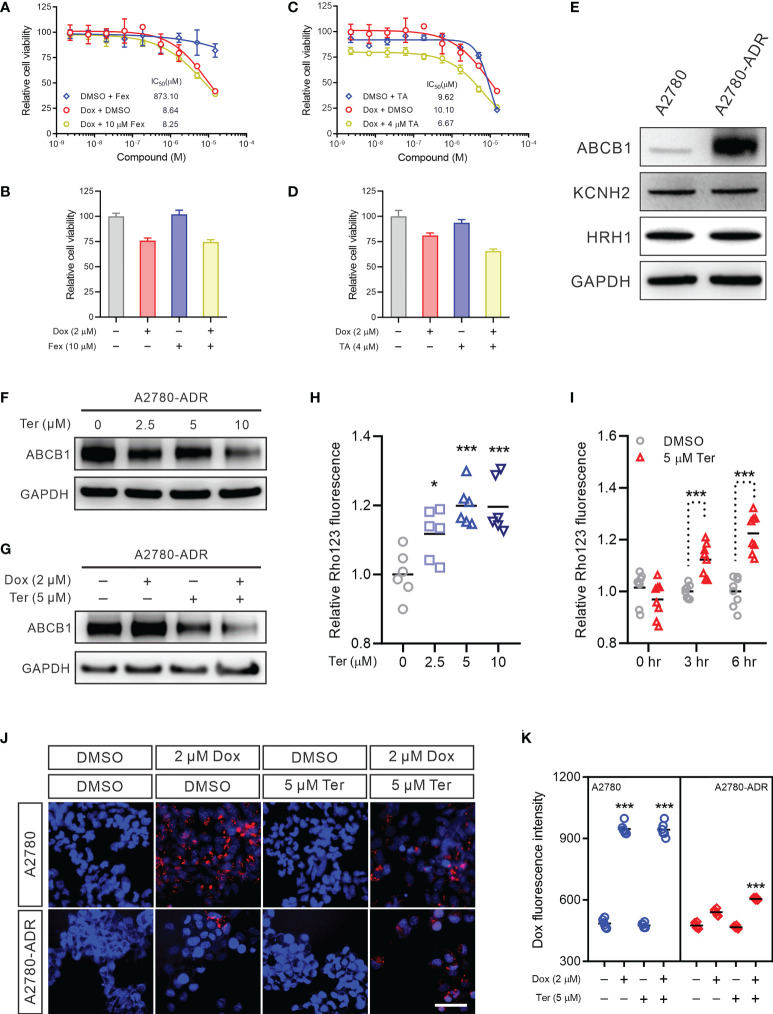
Terfenadine reverses MDR in ovarian cancer cells by repressing ABCB1 expression **(A)** Dose-response curves for doxorubicin (Dox) in the presence or absence of 10 μM fexofenadine (Fex) in MDR ovarian cancer cells. **(B)** Cell viability of MDR ovarian cancer cells treated with 2 µM doxorubicin (Dox), 10 µM fexofenadine (Fex), or both for 48 hours. DMSO was used as a non-treated control. **(C)** Dose-response curves of the MDR ovarian cancer cells to doxorubicin (Dox) in the presence or absence of 4 μM tannic acid (TA). **(D)** Cell viability of MDR ovarian cancer cells treated with 2 µM doxorubicin (Dox), 5 µM tannic acid (TA), or both for 48 hours. DMSO was used as a non-treated control. **(E)** Western blot analysis of hERG channel (KCNH2) and HRH1 in MDR ovarian cancer cell and its sensitive parental A2780 cell. **(F)** ABCB1 Western blot analysis in MDR cells treated with the indicated terfenadine (Ter) concentration. DMSO was used as a non-treated control. GAPDH was used as a loading control. **(G)** ABCB1 Western blot in MDR cells treated with 2 µM doxorubicin (Dox), 5 µM terfenadine (Ter), or both. DMSO was used as a non-treated control. GAPDH was used as a loading control. **(H)** Rho123 accumulation in MDR ovarian cancer cells treated with the indicated concentration of terfenadine (Ter) for 6 h. **(I)** Rho123 accumulation in MDR ovarian cancer cells treated for indicated time with 5 μM terfenadine (Ter). **(J, K)** Doxorubicin intracellular accumulation in MDR ovarian cancer cells or its parental sensitive A2780 cells treated for 6 hours with 2 µM doxorubicin (Dox), 5 µM terfenadine (Ter), or both. All values represent the mean ± SEM (n = 3 replicates). All values represent the mean ± SEM (n = 3 replicates). Western blot images were shown as one of three repeated experiments. Statistical analysis was carried out using a two-tailed t-test (* *p* < 0.05, *** *p <* 0.001).

To investigate the mechanism underlying this synergistic effect, we first examined two conventional terfenadine targets: the histamine H1 receptor (H1R) (37) and the human ether-a-go-go-related gene (hERG) channel (38). Terfenadine was reported as an antagonist of the H1R and is a prodrug that is converted to fexofenadine in the liver (37). Nevertheless, it was withdrawn from the market due to its ability to inhibit the hERG channel (38). The dose-response curve for doxorubicin as monotherapy was nearly comparable to the dose-response curve for doxorubicin in conjunction with the H1R-specific inhibitor fexofenadine (39) (**Figure 3A**). The CI for doxorubicin and fexofenadine was 0.9819, showing that their effect was additive, not synergistic (**Figures 3A, B**). In a second attempt, tannic acid (TA), a blocker of the hERG channel, was recruited (40). Even while TA decreased cell viability at higher concentrations, the dose-response curve for the combined therapy was similar to that of the doxorubicin treatment alone (**Figure 3C**). The CI value for the combination of doxorubicin and TA was 1.1867, showing that there was no synergy between the two drugs (**Figures 3C, D**). In addition, western blot revealed no difference in H1R and KCNH2 protein expression between A2780 and A2780-ADR cells, indicating that these proteins are not essential for MDR development (**Figure 3E** and **Figure S3**). These findings indicated that the synergistic effect of terfenadine and doxorubicin was not the result of terfenadine inhibiting the H1R or the hERG channel.

### Terfenadine increased the intracellular accumulation of doxorubicin in MDR ovarian cancer cells by repressing ABCB1

We questioned whether terfenadine impacts the expression or function of ABCB1 in these MDR ovarian cancer cells, as ABCB1 overexpression was essential for the chemoresistance of A2780-ADR. Indeed, terfenadine decreased the expression of the ABCB1 protein in a dose-dependent manner (**Figure 3F** and **Figure S4A**). The reduction of ABCB1 was also found following doxorubicin and terfenadine treatment (**Figure 3G** and **Figure S4B**). In addition, in the ABCB1 activity experiment, terfenadine boosted Rho123 accumulation dose-dependently, indicating the decreased cellular ABCB1 activity. The Rho123 signal achieved a plateau when the concentration exceeded 5 µM (**Figure 3H**). In a time-course study, the intracellular level of Rho123 continued to increase until terfenadine-induced apoptosis occurred (**Figure 3I**). Moreover, combination treatment significantly enhanced doxorubicin levels in A2780-ADR cells (**Figures 3J, K**). These findings imply that terfenadine decreased the expression and activity of the multidrug efflux pump ABCB1 in A2780-ADR cells, which resulted in the accumulation of doxorubicin and apoptosis.

### Calcium pathway was altered in MDR cells

**Figure 4:**
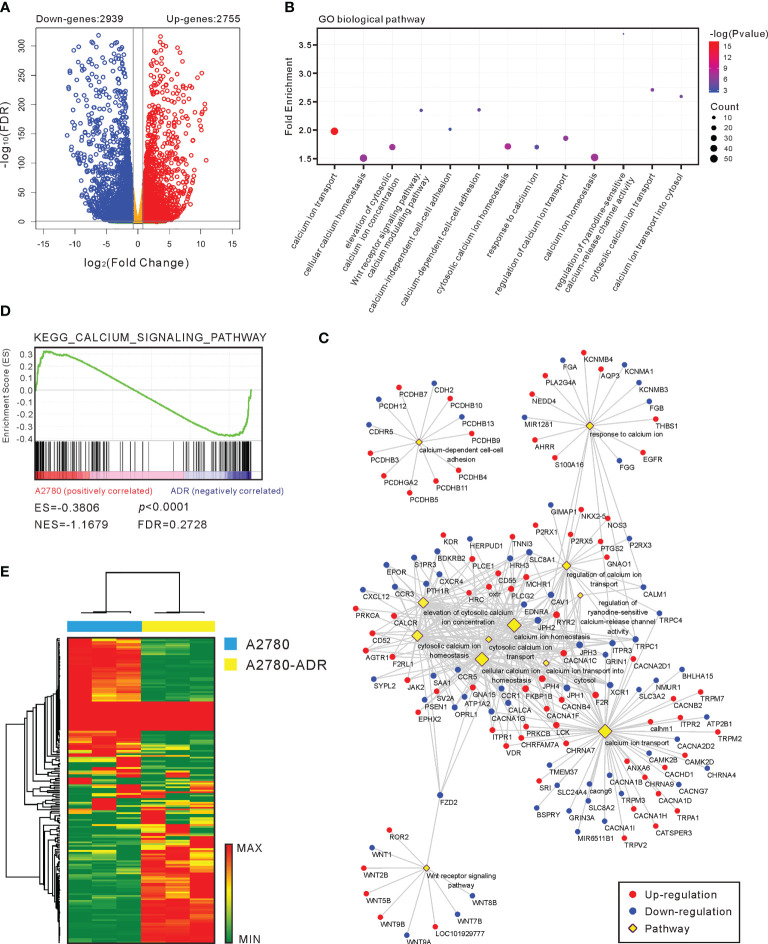
Calcium pathway is important in MDR generation. **(A)** A volcano plot of the genes that were up- and down-regulated in A2780-ADR cells versus A2780 cells. The genes are considered significant changes when the p-value is < 0.05 and the fold change is > 2-fold. **(B)** Gene ontology (GO) enrichment analysis revealed that 13 calcium-related biological pathways were activated in the A2780-ADR cell when compared to its parental A2780 cell. **(C)** Gene-Pathway network showed most of the DEGs were clustered in the processes of cytosolic calcium ion transport, homeostasis, and response. **(D)** The KEGG calcium signaling pathway (p < 0.0001) was also exhibited the significance in gene set enrichment analysis (GSEA). **(E)** Heatmap: the unsupervised hierarchical clustering showed 177 genes regarding calcium pathway showed the perfect separation in the GSEA.

To further investigate the potential targets and mechanisms of drug resistance, we used RNA-seq to assess the transcriptional differences between A2780 and A2780-ADR. A total of 5,694 genes with a fold change of > 2 and a p-value < 0.05 were identified as differentially expressed genes (DEGs) (**Figure 4A**), including 2,755 genes that were over-expressed and 2,939 genes that were under-expressed in the A2780-ADR cells. The gene ontology (GO) enrichment study revealed 13 calcium-related biological processes which caught our attention (**Figure 4B**). The Gene-Pathway network showed that the majority of DEGs were clustered in the cytosolic calcium ion transport, homeostasis, and response processes (**Figure 4C**), indicating this MDR cell line possessed abnormal calcium signaling.

In addition to the preliminary analysis, a gene set enrichment analysis (GSEA) was conducted to identify probable biological pathway enrichment from the Kyoto Encyclopedia of Genes and Genomes (KEGG) pathway database. This study uncovered the difference in the KEGG calcium signaling pathway (p<0.0001) as well (**Figure 4D**). Using all 177 genes involved in the calcium pathway, unsupervised hierarchical clustering revealed a clear separation of these two cell types (**Figure 4E**), demonstrating a major modification in the calcium homeostasis of MDR cells. Collectively, these findings suggest that the calcium signaling pathway is associated with the MDR phenotype in the A2780-ADR cells.

### Terfenadine overcomes MDR by inhibiting the CAMK2/CREB1 pathway

**Figure 5:**
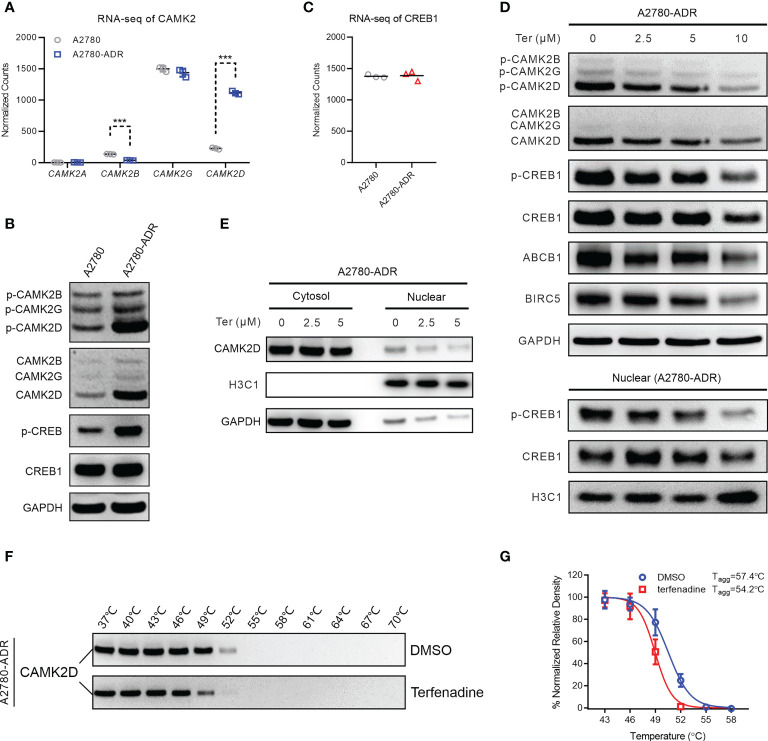
Terfenadine overcomes MDR in ovarian cancer cells by inhibiting the CAMK2/CREB-mediated pathway. **(A)** Normalized read counts of the CAMK2 family in MDR ovarian cancer cells and its parental sensitive ovarian cells (A2780) obtained in the RNA-seq analysis. **(B)** Western blot analysis of p-CAMK2 (T286), CAMK2 (pan), p-CREB1 (s133), and CREB1 in MDR sensitive ovarian cells (A2780). GAPDH was used as the loading control. **(C)** RNA-seq normalized read counts of CREB1 in MDR ovarian cancer cells and its parental sensitive ovarian cancer cells A2780. **(D)** Upper panel: Western blot analysis of p-CAMKII (T286), CAMKII (pan), p-CREB1 (s133), CREB1, ABCB1, and BIRC5 in MDR ovarian cancer cells treated for 24 hours with terfenadine (Ter). GAPDH was used as the loading control. Lower panel: Western blot analysis of CREB1 and p-CREB1 (s133) in the nucleus of MDR ovarian cancer cells treated with terfenadine (Ter) for 6 h. Histone H3 (H3C1) was used as the loading control. **(E)** Western blot analysis of CAMK2D in the cytosol or nucleus of terfenadine (Ter)-treated MDR ovarian cancer cells for 6 hours. GAPDH and H3C1 were used as the loading controls for cytosol protein and nucleus protein, respectively. **(F**, **G)** Cellular thermal shift assay (CETSA) for the binding of terfenadine to CAMKIID in MDR ovarian cancer cell lysate. **(F)** Representative western blot images for the CESTA. **(G)** T_agg_ curves of CaMKIID in MDR ovarian cancer cells in the presence of DMSO or 600 µM of terfenadine. All statistical analysis was performed using a two-tailed t-test (*** *p* < 0.001).

Among the proteins implicated in the calcium signaling pathway, RNA-seq data revealed dysregulation of calcium/calmodulin-dependent protein kinase II (CaMK2) members. Specifically, *CAMK2D* is highly up-regulated, and *CAMK2B* is down-regulated (**Figure 5A**). The rise of CAMK2D and its active form, phosphorylated-CAMK2D (T286), in A2780-ADR cells was confirmed by Western blotting (**Figure 5B** and **Figure S5A**). In addition, we detected an increase in the phosphorylation of CREB1 at S133, although RNA and protein levels remained unchanged (**Figures 5B, C** and **Figure S5A**). As revealed by these results, they indicated the CAMK2/CREB1 pathway was overactive in the A2780-ADR cells.

To determine if terfenadine blocked the CAMK2/CREB1 pathway, the expression of related proteins was measured following terfenadine administration. After 24 hours of treatment, dose-dependent reductions in CAMK2D and phosphorylated CAMK2D were observed. Meanwhile, CREB1, ABCB1, and baculoviral inhibitor of apoptosis repeat-containing 5 (BIRC5) were also reduced (**Figure 5D** and **Figure S5B**). The protein BIRC5, also known as survivin, suppresses apoptosis by inhibiting caspase activation. As CREB1 is a transcription factor located in the nuclei and is activated by direct binding of CAMK2, we also examined their levels in the nuclei and found that terfenadine dose-dependently decreased the CAMK2D and the phosphorylated CREB1 in the nuclei (**Figures 5D, E** and **Figures S6A, B**), indicating a decrease in the activating and nuclear entry of CAMK2D in the presence of terfenadine.

A cellular thermal shift assay (CETSA) was conducted to assess if terfenadine directly binds to CAMK2D to prevent its activation. CAMK2D’s apparent aggregation temperature (T_agg_) was evaluated in the absence or presence of terfenadine in A2780-ADR cell lysates (**Figure 5F** and **Figure S6C**). The best-fit curve for the terfenadine-treated group shifted significantly from that of the DMSO control (p< 0.001). **Figure 5G** shows that terfenadine reduced the T_agg_ of CaMK2D protein from 57.4 to 54.2°C, indicating that it thermally destabilized CAMK2D. Together with the other studies demonstrating that CREB1 regulates ABCB1 expression (41, 42), our findings suggest that terfenadine may prevent cells from apoptosis by regulating the Ca^2+^-mediated CAMK2/CREB1 pathway through binding directly to CAMK2D, thereby causing its destabilization in cells and reducing the activation of CREB1 and subsequent ABCB1 expression.

### CAMK2/CREB1 pathway is the promising therapeutics target for the ABCB1 mediated MDR of ovarian cancer

**Figure 6:**
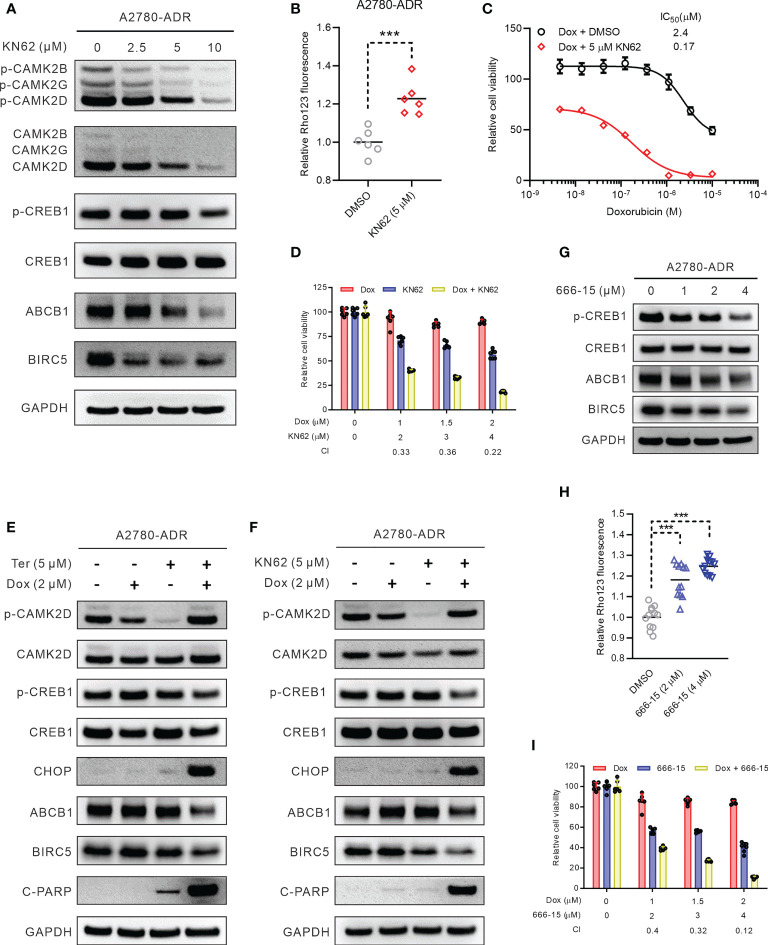
Inhibiting the CAMK2/CREB pathway reversed MDR in ovarian cancer cells. **(A)** Western blot of p-CAMK2 (T286), CAMK2 (pan), ABCB1, p-CREB1 (s133), CREB1, and BIRC5 in KN62-treated MDR ovarian cancer cells for 24 h. **(B)** Rho123 accumulation in MDR ovarian cancer cells treated for 6 h with 5 µM KN62. DMSO was used as non-treated control. Statistical analysis was performed using a two-tailed t-test (*** p < 0.001). **(C)** Dose-response curves of MDR ovarian cancer cells to doxorubicin (Dox) in the presence or absence of 5 µM KN62. **(D)** Synergistic effects of KN62 and doxorubicin (Dox) on MDR ovarian cancer cell killing. The bar graph shows the cell viability of MDR ovarian cancer cells treated for 48 h with doxorubicin, KN62, or both. Calculated CI values are presented below the plots. **(E)** Western blot of p-CAMK2 (T286), CAMK2 (pan), ABCB1, p-CREB1, CREB1, c-PARP, CHOP, and BIRC5 in MDR ovarian cancer cells treated for 24 h with 2 µM doxorubicin (Dox), 5 µM terfenadine (Ter), or both. **(F)** Western blot of p-CAMK2 (T286), CAMK2 (pan), ABCB1, p-CREB1, CREB1, c-PARP, CHOP, and BIRC5 in MDR ovarian cancer cells treated for 24 h with 2 µM doxorubicin (Dox), 5 µM KN62, or both. **(G)** Western blot of ABCB1, p-CREB1 (s133), CREB1, and BIRC5 in MDR ovarian cancer cells treated for 24 h with 666-15. **(H)** Rho123 accumulation in MDR ovarian cancer cells treated for 6 h with 666-15. DMSO was used as non-treated control. Statistical analysis was performed using a one-way ANOVA with Tukey’s HSD correction (*** p < 0.001). **(I)** Synergistic effects of 666-15 and doxorubicin (Dox) on MDR ovarian cancer cell killing. The bar graph shows the cell viability of MDR ovarian cancer cells treated for 48 h with doxorubicin, 666-15, or both. Calculated CI values are presented below the plots.

To confirm further that CAMK2D was a target for MDR combination therapy, we recruited KN62, a CaMK2 specific inhibitor, for our investigation (43). KN62 reduced the expression and activity of ABCB1 in A2780-ADR cells, consistent with the terfenadine therapy (**Figures 6A, B** and **Figure S7A**). Moreover, KN62 inhibited the expression of BIRC5 and the phosphorylation of both CAMK2D and CREB1 in A2780-ADR cells (**Figure 6A**). The IC_50_ of doxorubicin dropped from 2.4 µM (doxorubicin alone) to 0.17 µM (doxorubicin paired with 5 µM KN62) in A2780-ADR cells when KN62 was administered in combination with doxorubicin (**Figure 6C**). This was a synergistic combination (CI < 1) (**Figure 6D**). Notably, KN62 had the same effect as terfenadine in the combination with doxorubicin, decreasing ABCB1 and BIRC5 expression and increasing cleaved PARP (**Figures 6E, F**). Although the phosphorylated CAMK2D was lowered in either terfenadine or KN62 treatment alone, there was no difference in CAMK2D phosphorylation in combination treatments with either of them with doxorubicin. In contrast, CREB1 phosphorylation remained lower in the combined treatment, indicating that CAMK2 inhibitors repressed the CAMK2/CREB1 pathway (**Figures 6E. F** and **Figures S8A, B**). Furthermore, KN93, another CAMK2-specific inhibitor, reduced ABCB1 activity and increased doxorubicin-induced cell death in A2780-ADR cells (**Figures S9A, B**). These findings suggest that inhibiting CAMK2 could resensitize MDR cells to doxorubicin.

To determine if blocking CREB1 would similarly resensitize MDR cells to doxorubicin, A2780-ADR cells were treated with the selective CREB1 inhibitor 3i (also known as 666-15 (44). Consistent with the CAMK2 inhibitors, ABCB1 and BIRC5, along with CREB1 phosphorylation, decreased after treatment with 666-15 (**Figure 6G** and **Figure S7B**). Under the 666-15 treatment, the total ABCB1 activity similarly dropped in a dose-dependent manner (**Figure 6H**). In addition, the combination of doxorubicin and 666-15 killed MDR cells A2781-ADR synergistically (CI < 1) (**Figure 6I**). Together, inhibition of the Ca^2+^ mediated CAMK2D/CREB1 pathway appears to be a promising therapeutic target for doxorubicin resensitization in ABCB1-mediated MDR ovarian cancer.

## Discussion

Resistance to chemotherapy, whether inherited or acquired, is a significant obstacle in cancer treatment. Several mechanisms of drug resistance have been postulated, with the multiplication and expression of phosphorylated ABCB1 protein, an energy-dependent drug efflux pump, being one of the most extensively investigated (45). Studies *in vitro* have demonstrated that high levels of ABCB1 expression are associated with MDR in multiple cell lines and that the degree of overexpression correlates with the amount of resistance (46). Research on patients with ovarian cancer has found that high levels of ABCB1 expression are inversely related to chemotherapy response and progression-free survival (47). Consequently, ovarian cancer patients continue to be in need of a therapeutic for effectively overcoming MDR. Using qHTCS, this work found a group of doxorubicin potentiators in an ABCB1-mediated MDR ovarian cancer cell line. Among these, we demonstrated that terfenadine restored the activity of doxorubicin by inhibiting the CAMK2/CREB1 pathway, resulting in decreased expression of ABCB1 and BIRC5. In addition, inhibiting the CAMK2/CREB1 pathway resensitized MDR ovarian cancer cells to not only doxorubicin but also paclitaxel and topotecan, which are clinically employed to treat ovarian cancer (48).

Terfenadine has been shown to restore the activity of doxorubicin in the MCF-7/ADR human breast cancer cells and the L1210/VMDRC.06 murine leukemia cells (49), and the activity of epirubicin in killing drug-resistant non-small cell lung cancer (20). In spite of this, the target and mechanism by which terfenadine restores chemotherapeutic activity in MDR cancer cells remain unknown. Notably, neither H1R nor hERG inhibitors were able to duplicate the synergistic effects of terfenadine on the MDR cancer cells, indicating that other biological mechanisms may be involved in the reversal of chemosensitivity. To investigate the unique target of terfenadine in combinational chemotherapy for MDR cancer, the global gene expression of doxorubicin-sensitive and -resistant cell lines was profiled using RNA sequence. Importantly, calcium signaling-related pathways were shown to be aberrantly regulated in MDR cells, indicating that calcium homeostasis was disrupted. Indeed, our work demonstrated the abnormal expression of CAMK2 family members, particularly CAMK2D, which is dramatically overexpressed in MDR cells, and terfenadine treatment inhibits the CAMK2D phosphorylation in a manner comparable to that of the CAMK2 inhibitor KN62.

Intriguingly, terfenadine has been related to a decrease in calcium influx caused by L-type calcium channels (LTCC) activation in rat cerebellar neurons and human atrial myocytes (22, 23), showing terfenadine can regulate intracellular calcium homeostasis. However, the target of terfenadine for this function remains unclear. Moreover, activation of CAMK2 can further activate LTCC by binding to and phosphorylating the COOH terminus of LTCC (50, 51). Using the CETSA assay, we demonstrated the direct binding of terfenadine to CAMK2D in our study, as indicated by a protein melting curve shift after the addition of terfenadine to the cell lysate. Interestingly, the melting curve of the CAMK2D protein was shifted to the right in the presence of terfenadine, indicating instability of the CAMK2D protein upon heating when bound to terfenadine. As equilibrium binding ligands typically increase protein thermal stability by a factor proportionate to the concentration and affinity of the ligand, the CETSA assay will typically demonstrate a leftward change in the melting curve of the protein (52). However, multiple situations have been reported experimentally in which equilibrium-binding ligands destabilize proteins, i.e., decrease the melting temperature of the protein by an amount proportionate to the ligand’s concentration and affinity (53, 54). This type of protein instability may cause aggregation and degradation of target proteins in cells, resulting in further protein reduction. In our study, we demonstrated that terfenadine administration lowered CAMK2 protein in a dose-dependent manner. Based on our findings, it is possible to speculate that terfenadine’s inhibition of CAMK2 protein leads to the deactivation of LTCC, thereby reducing calcium influx in neurons and myocytes.

Unfortunately, terfenadine has been linked to cardiac death in at least 125 and 14 cases in the United States and United Kingdom, respectively (55), and the Food and Drug Administration (FDA) recommended its removal from the market in 1997 due to its pro-arrhythmic risk for long QT-related Torsades de Pointes (TdPs) (56, 57). Although numerous structural derivatives with a relatively low toxicity profile, such as fexofenadine (58), have been developed, their activity and target for MDR cancer treatment have yet to be investigated. Therefore, it is preferable to identify pharmacologically accessible downstream targets in this calcium cascade for MDR treatment. In recent years, CAMK2 has garnered a great deal of attention for its pivotal role in the arrhythmias of chronic illness (59). The isoform-specific inhibitor of CAMK2D (the main cardiac isoform of CAMK2) could be used to target the cardiac-specific pathology of autonomously activated CAMK2 in diabetes (60), while avoiding off-target effects in other tissues, such as α and β isoforms of CAMK2, and disruption of memory formation in the hippocampus (61). A recent clinical trial revealed that the CAMK2 inhibitor appears to be well accepted and safe among patients (62), suggesting that it should pave the way for future development of CAMK2 inhibitors in other conditions, such as the treatment of MDR cancer patients.

Considering that CAMK2 activation can phosphorylate and activate CREB1 (63) and that phosphorylated CREB1 binds to the CRE binding site in the ABCB1 promoter and promotes ABCB1 expression (64), CAMK2 activation will induce ABCB1 expression in cancer cells, resulting in MDR. Therefore, reducing CREB1 activity is an additional promising MDR cancer therapeutic target. In fact, CREB has already been identified as a candidate for oncogenic signaling in a variety of tumor types (65), particularly in leukemia and glioma (66, 67). In the current work, an aberrant increase in CREB1 phosphorylation was observed in MDR cells, and inhibition of CREB1 decreased ABCB1 expression and activity, indicating that CREB1 is a viable target for MDR reversal in cancer therapy. Despite the recent developments, CREB inhibitors are exclusively used in preclinical research. The lack of pharmacokinetic and pharmacodynamic responses, as well as toxicity reports, makes it unlikely that any of them will be used in clinical practice currently, despite the fact that some of them look to be highly promising. In addition, the use of CREB inhibitors has been hampered by numerous limitations, such as lower bioactivity in living systems and off-target binding. This necessitates a more comprehensive characterization and development prior to clinical application.

**Figure 7:**
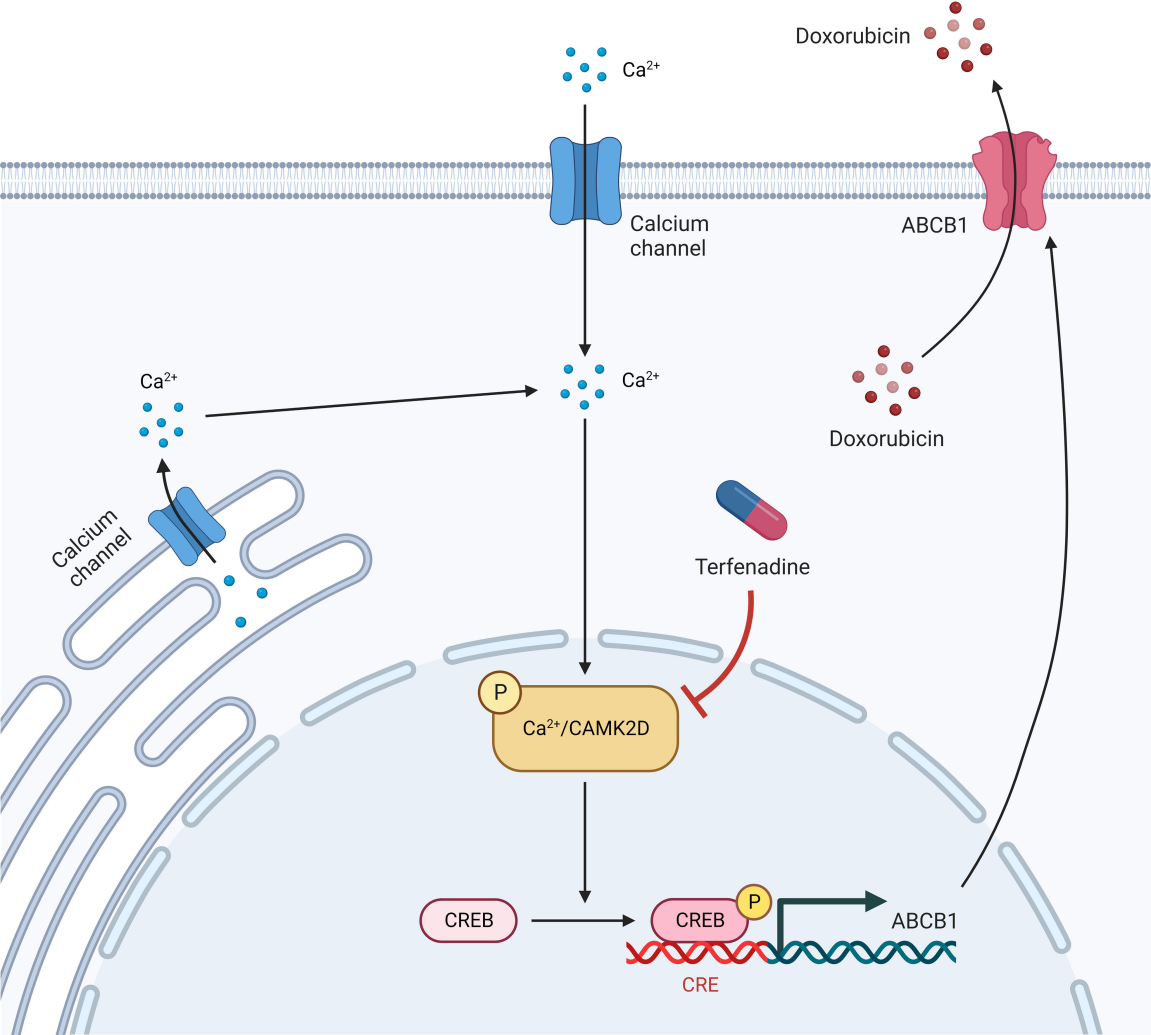
An illustration of terfenadine function in combinational treatment with doxorubicin.

In this study, we reported that the CAMK2/CREB pathway, particularly CAMK2D, is a promising target for reversing ABCB1-mediated drug resistance in ovarian cancer (**Figure 7**). However, the *in vivo* activity of their inhibitors requires further investigation. Additionally, we demonstrated once more that integrating qHTCS and gene expression data is an effective approach for identifying novel agents with combinational effects and their underlying mechanisms.

## Data availability statement

The datasets presented in this study can be found in online repositories. The names of the repository/repositories and accession number(s) can be found on: https://www.ncbi.nlm.nih.gov/, GSE177038.

## Author contributions

WL and WZ conceived the research and designed the study. WH, SY, NS, and WS performed the experiments. WH, SY, Y-SC, MS and JB analyzed the data. SY, Y-SC and WZ wrote, reviewed, and edited the manuscript. All authors contributed to the article and approved the submitted version.

## Funding

This work was partially supported by the intramural research programs of the National Center for Advancing Translational Sciences, National Institutes of Health, and the Natural Science foundation of Zhejiang Province (LY19H160046 to WH). This work was also supported by grants from National Natural Science Foundation of China (81974403 to NS).

## Acknowledgments

The authors thank the compound management group at NCATS, NIH for their professional support and Dr. DeeAnn Visk, a medical writer and editor, for editing the manuscript.

## Conflict of interest

The authors declare that the research was conducted in the absence of any commercial or financial relationships that could be construed as a potential conflict of interest.

## Publisher’s note

All claims expressed in this article are solely those of the authors and do not necessarily represent those of their affiliated organizations, or those of the publisher, the editors and the reviewers. Any product that may be evaluated in this article, or claim that may be made by its manufacturer, is not guaranteed or endorsed by the publisher.
