# Supplementary figures and images for: Terfenadine resensitizes doxorubicin activity in drug-resistant ovarian cancer cells via an inhibition of CaMKII/CREB1 mediated ABCB1 expression
Source: Front Oncol. 2022 Nov 10;12:1068443. doi: 10.3389/fonc.2022.1068443 (PMC9684669; doi:10.3389/fonc.2022.1068443)

## Slide 1
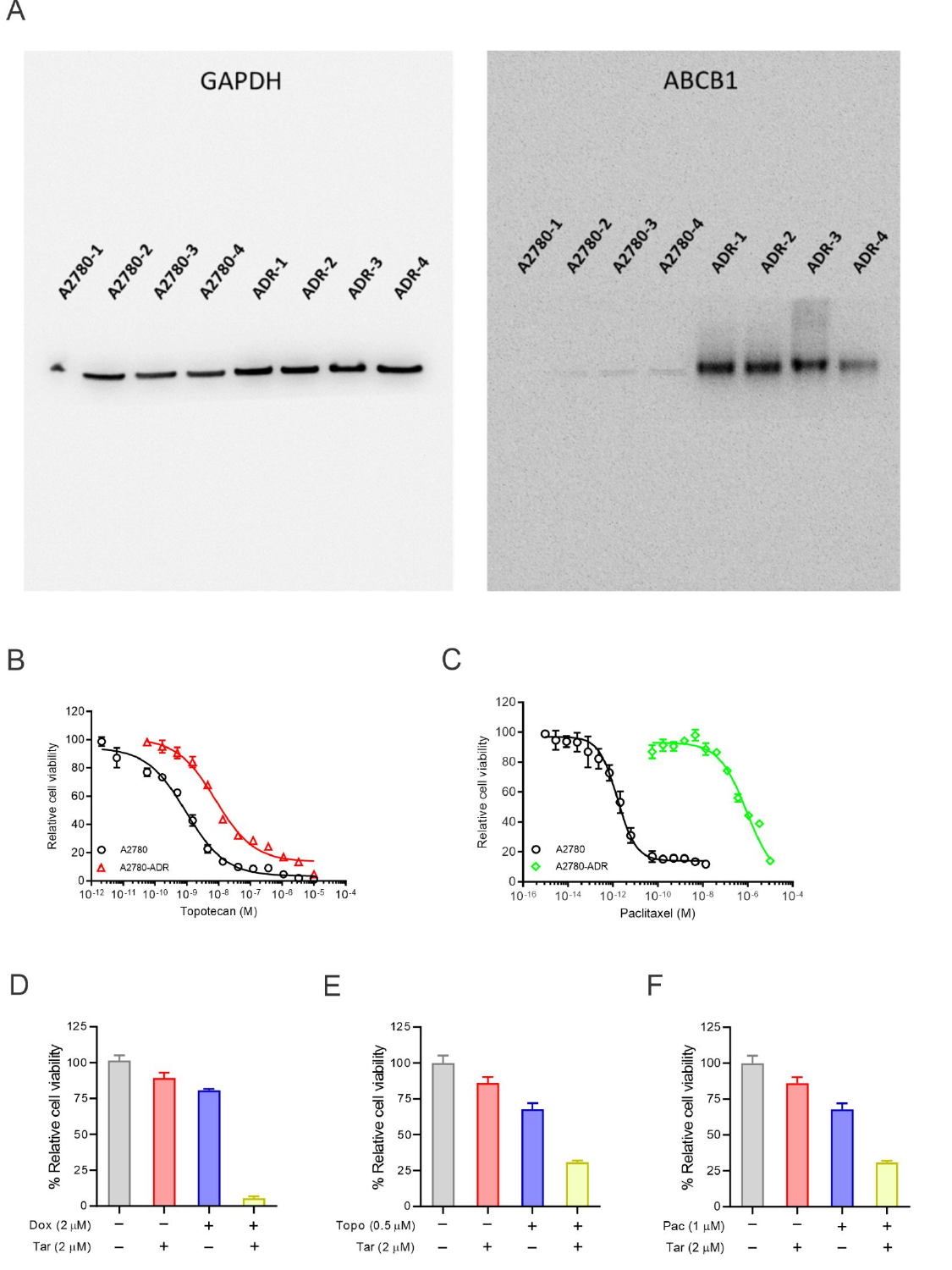

## Slide 2
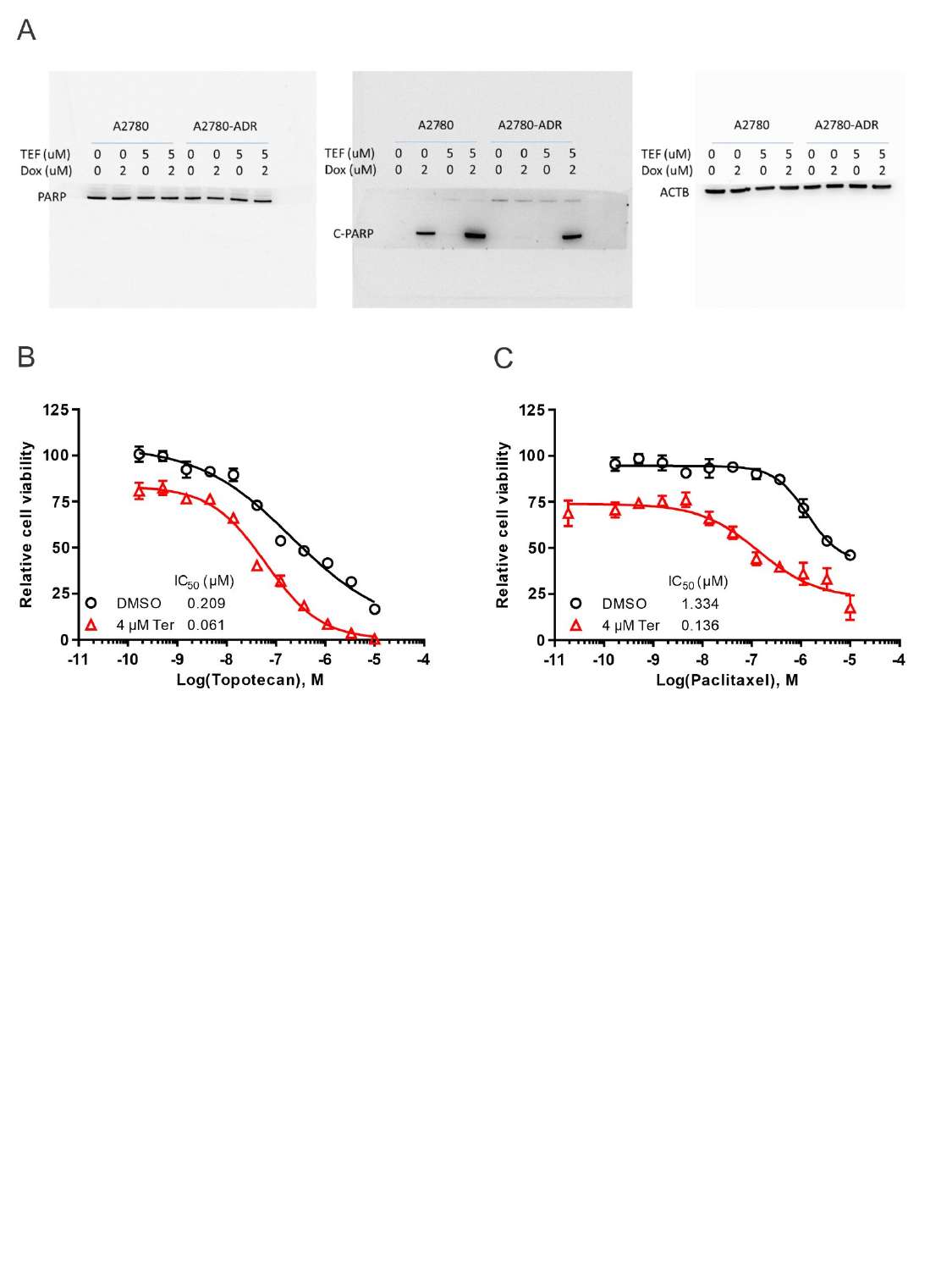

## Slide 3
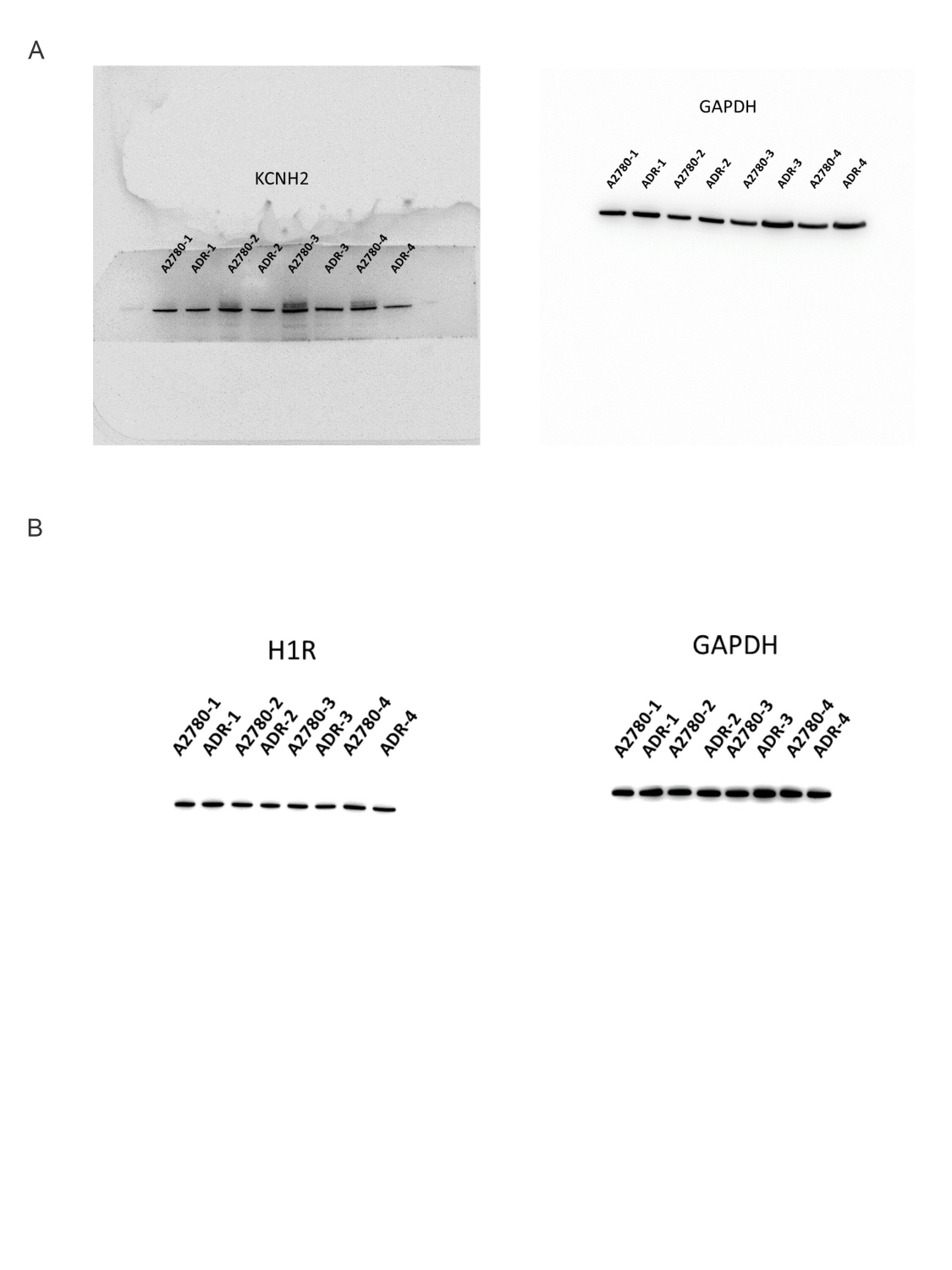

## Slide 4
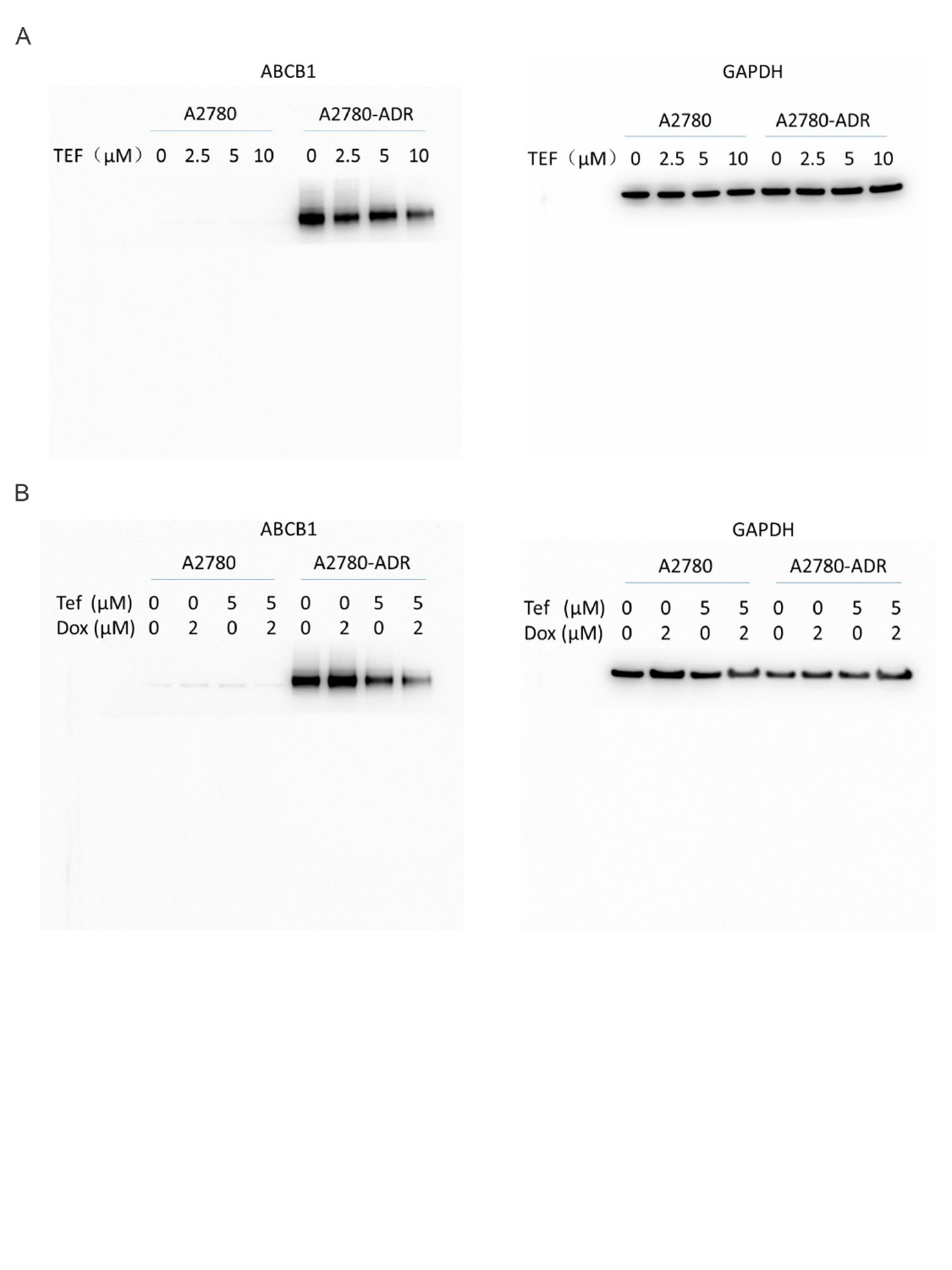

## Slide 5
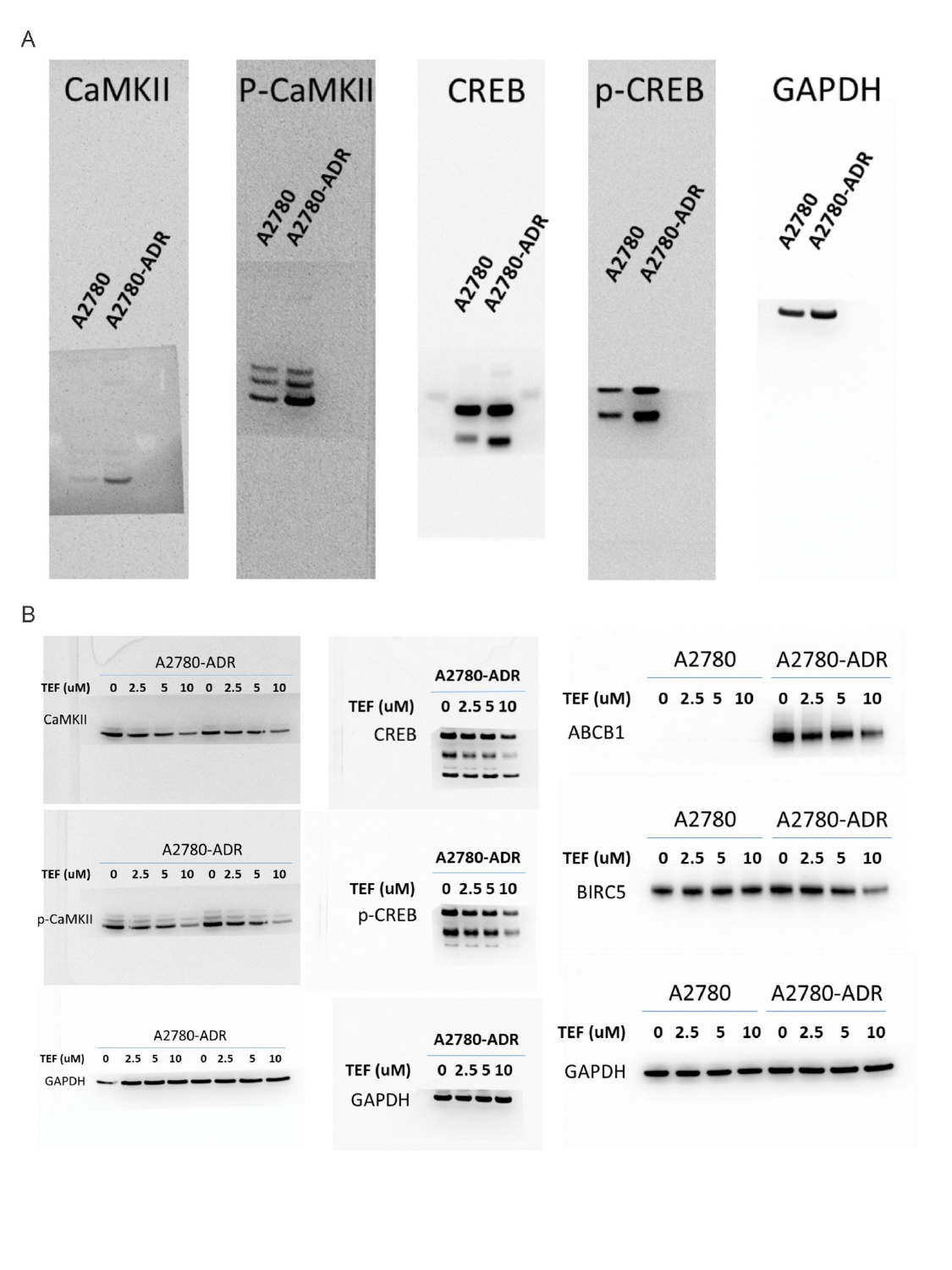

## Slide 6
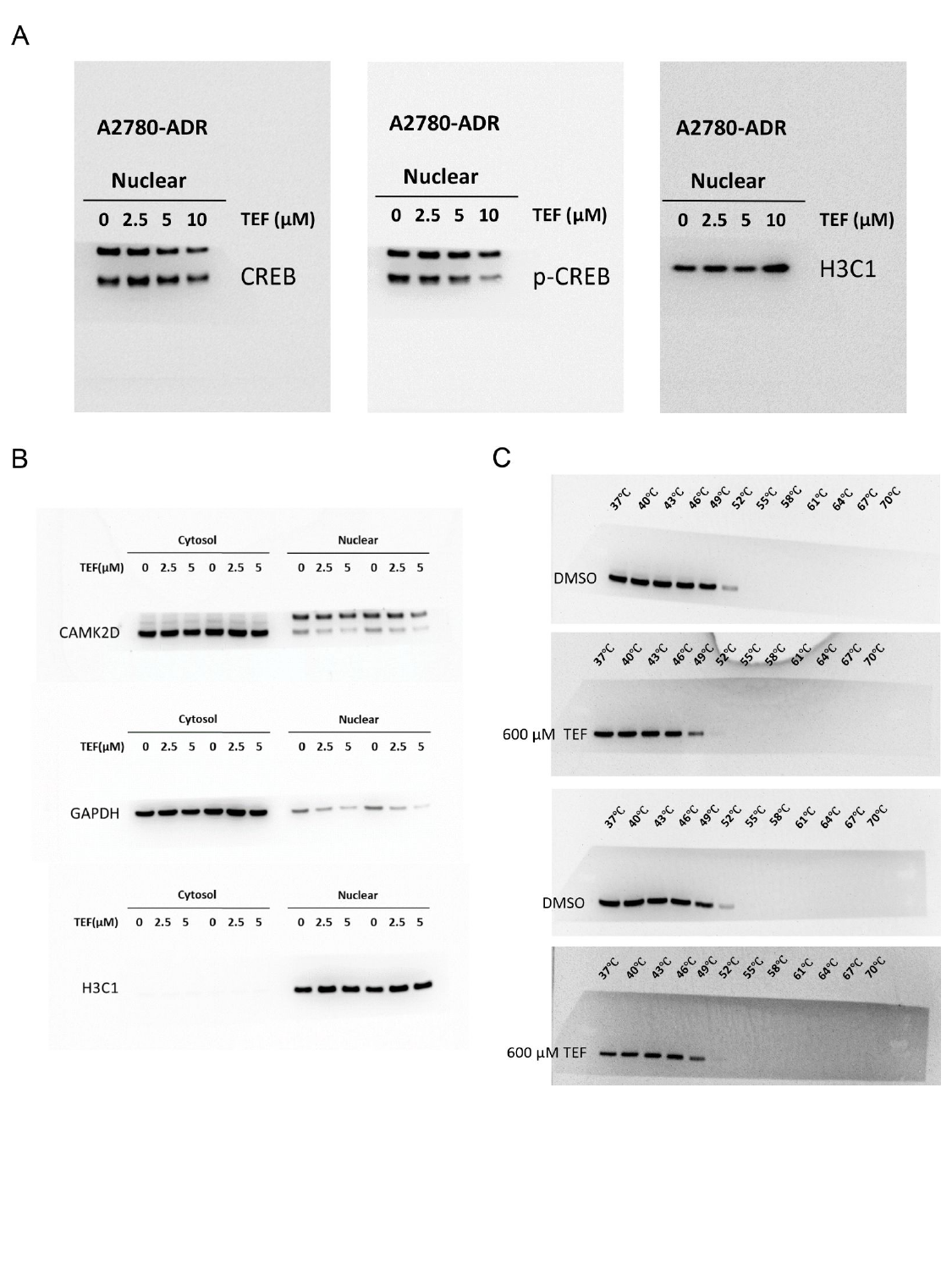

## Slide 7
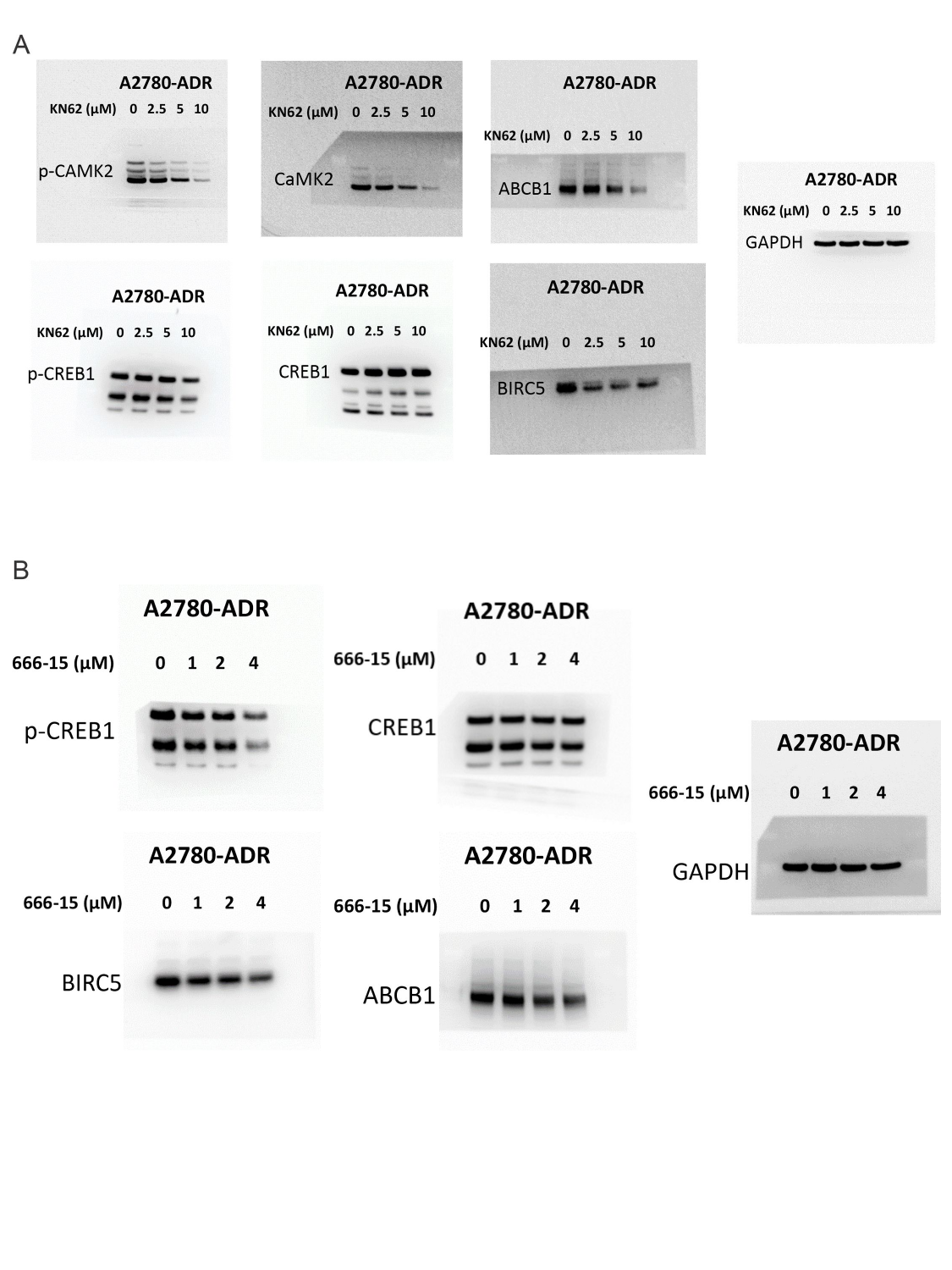

## Slide 8
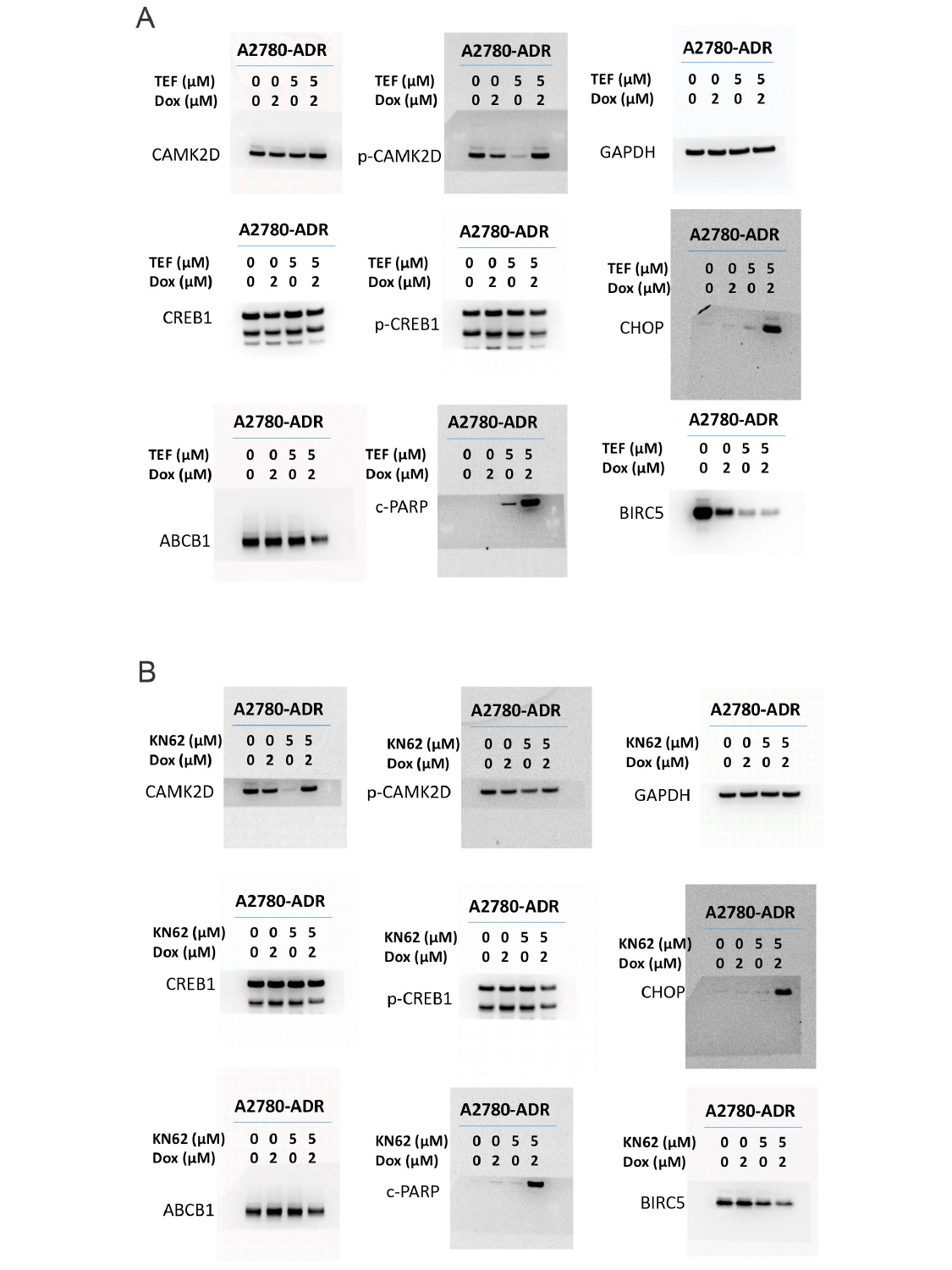

## Slide 9
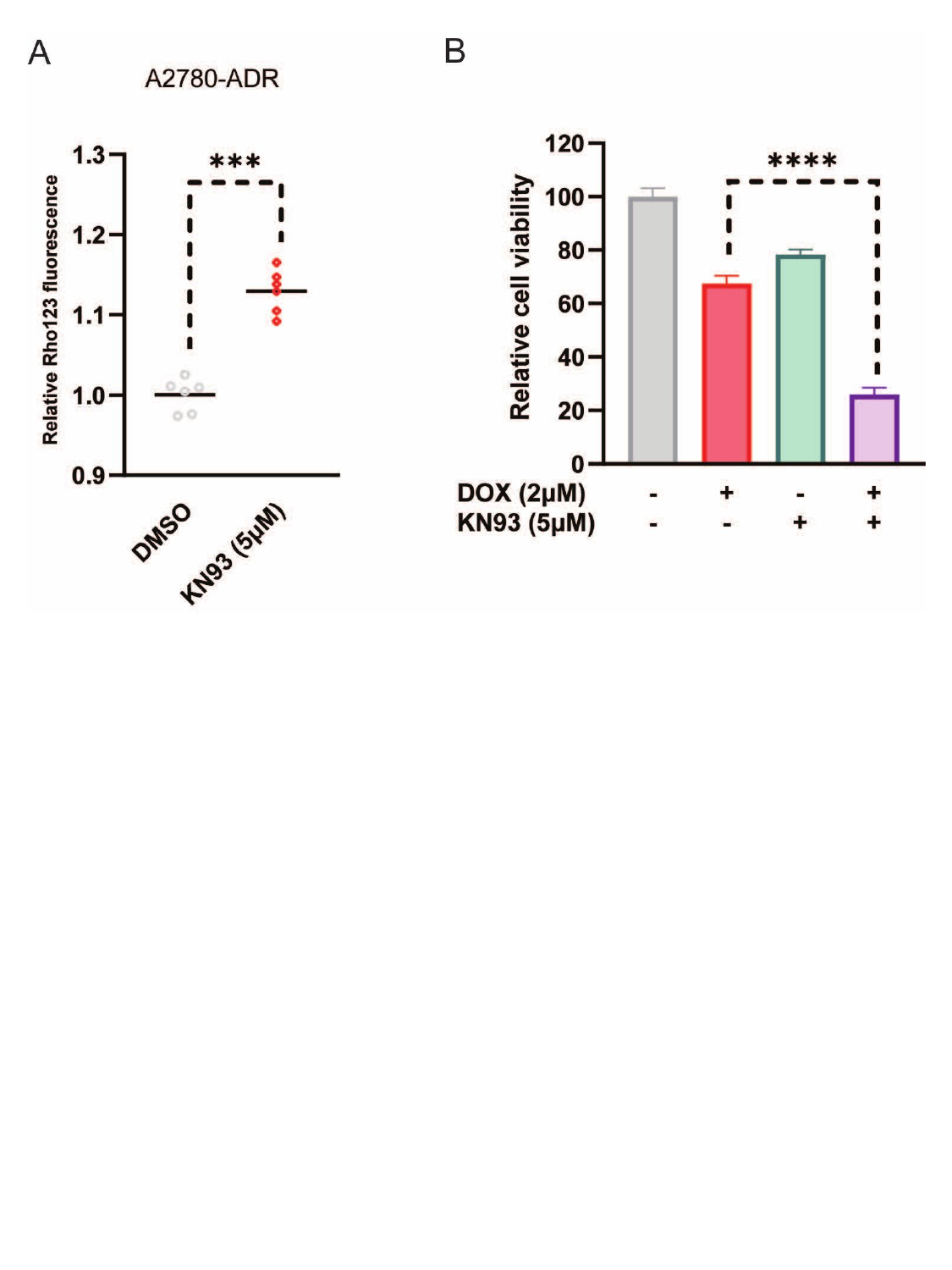

Supplement: Supplementary file 1 [file Presentation_1.pptx]
