# Supplementary material for: Terfenadine resensitizes doxorubicin activity in drug-resistant ovarian cancer cells via an inhibition of CaMKII/CREB1 mediated ABCB1 expression
Source: Front Oncol. 2022 Nov 10;12:1068443. doi: 10.3389/fonc.2022.1068443 (PMC9684669; doi:10.3389/fonc.2022.1068443)
Supplement: Supplementary file 2 [file Table_1.docx]

Table S1 summary of antibodies used in experiments

| Name | Isotype | Vender | Catalog No. | Dilution factor |
| --- | --- | --- | --- | --- |
| p-CaMKII-α(T286) | Rabbit | Cell Signaling Technology | 12716s | 1:1000 |
| CaMKII(pan) | Rabbit | Cell Signaling Technology | 4436s | 1:1000 |
| C-caspase3 | Rabbit | Cell Signaling Technology | 9664s | 1:1000 |
| C-PARP | Rabbit | Cell Signaling Technology | 5625s | 1:1000 |
| P-CREB | Rabbit | Cell Signaling Technology | 9198s | 1:1000 |
| CREB | Rabbit | Cell Signaling Technology | 9197s | 1:1000 |
| GAPDH | Rabbit | Cell Signaling Technology | 5174s | 1:1000 |
| ACTB | Mouse | Cell Signaling Technology | 3700s | 1:1000 |
| ABCB1 | Rabbit | Cell Signaling Technology | 12683s | 1:1000 |
| Survivin | Rabbit | Cell Signaling Technology | 2808s | 1:1000 |
| hERG | Rabbit | Cell Signaling Technology | 12889s | 1:1000 |
| H1R | Rabbit | Abcam | ab154158 | 1:1000 |
| Anti-rabbit IgG, HRP | Rabbit | Cell Signaling Technology | 7074s | 1:5000 |
| Anti-mouse IgG, HRP | Mouse | Cell Signaling Technology | 7076s | 1:5000 |
