# Supplementary material for: Terfenadine resensitizes doxorubicin activity in drug-resistant ovarian cancer cells via an inhibition of CaMKII/CREB1 mediated ABCB1 expression
Source: Front Oncol. 2022 Nov 10;12:1068443. doi: 10.3389/fonc.2022.1068443 (PMC9684669; doi:10.3389/fonc.2022.1068443)
Supplement: Supplementary file 3 [file Table_2.docx]

Table S2. Compounds of secondary hits by qHTCS

| Name | Primary mechanism of action | 100 nM Dox IC_50_ (µM) | 25 µM Dox IC_50_ (µM) | Fold of IC_50_ Change |
| --- | --- | --- | --- | --- |
| Devazepide | CCK receptor antagonist | null | 9.35 | null |
| Telithromycin | Ketolide antibiotic | null | 0.74 | null |
| Vardenafil | Phosphodiesterase V (PDE5) inhibitor | null | 1.05 | null |
| Tetrandrine | Calcium Channel Blocker | 33.17 | 0.83 | 39.81 |
| U-74389G maleate | Inhibitor of iron-dependent lipid peroxidation | 23.49 | 1.32 | 17.78 |
| Nilotinib | Bcr-Abl Kinase Inhibitor | 22.39 | 1.12 | 19.95 |
| GW 843682X | Polo-like Kinase-1 (Plk-1) Inhibitor | 19.14 | 2.70 | 7.08 |
| ITD-1 | TGF-b Inhibitor | 18.65 | 2.35 | 7.94 |
| Triciribine phosphate | PKB/Akt Inhibitor | 18.65 | 2.64 | 7.08 |
| Ketoconazole | Aromatase Inhibitor | 18.65 | 1.87 | 10.00 |
| Cortivazol | Glucocorticoid Receptor (GR) Agonist | 18.65 | 1.32 | 14.13 |
| Monatepil | Calcium Channel Blocker | 18.65 | 2.64 | 7.08 |
| Ponatinib | FGFR Inhibitor | 17.06 | 2.15 | 7.94 |
| Apatinib | VEGFR-2 inhibitor | 16.63 | 3.72 | 4.47 |
| CHIR-124 | Chk1 Inhibitor | 13.21 | 3.32 | 3.98 |
| KUC107871N-04 | p97 ATPase Inhibitor | 9.35 | 1.18 | 7.94 |
| Terfenadine | H1R inhibitor | 9.35 | 1.05 | 8.91 |
| CUDC-907 | PI3K Inhibitor | 5.90 | 1.32 | 4.47 |
| Gimatecan | DNA topoisomerase I inhibitor | 4.18 | 0.37 | 11.22 |
| NCGC00161703 | NF-kB/AP-1 activation inhibitor | 3.72 | 0.47 | 7.94 |
| BAG-956 | PI3K/PDK1 Inhibitor | 3.40 | 0.34 | 10.00 |
| Camptothecin | DNA Topoisomerase I Inhibitors | 2.64 | 0.66 | 3.98 |
| INK 128 | mTORC1/2 inhibitor | 1.32 | 0.93 | 1.41 |
| Triptolide | Inhibition of RNA polymerase II mediated transcription | 0.07 | 0.01 | 6.31 |
